# On the Decrease of Disease Effected by the Progress of Civilization

**Published:** 1845-11

**Authors:** C. F. H. Marx, R. Willis

**Affiliations:** London; London


*On the Decrease of Disease effected by the Progress of Civilization.* By C. F. H. Marx, M. D. *and* R. Willis, M. D. *&c. London*, 1844.

With the contents of this little volume we have already been made acquainted, as they appeared very recently at different times in the pages of the London Medical Gazette. We think Dr. Willis has done good service, not only to his professional brethren but to the public generally, in bringing it out in its present form. We fully concur with the Doctor in regretting that physicians have no place in the body politic, and in thinking that it would be well for humanity if they had. It cannot be denied that, since the revival of letters in Europe, medical men have been foremost in every undertaking whose object has been to extend the boundaries of knowledge and to exalt mankind. No class of men know half so much of the wants and the wishes, of the joys and the sorrows, of the community—they it is, who are the friends and comforters, in adversity especially, of persons in every grade of life—from the sovereign and the peer to the wretched outcast in the street. They it is, who follow in the field through thickest of the fire, not that they may aid destruction in her work, but God-like, they may staunch the wounds she makes. "Oh!" feelingly exclaims Dr. W. "let society cherish and exalt its medical community; let it become aware that if *science* cannot aid it in its struggles with disease, neither can *ignorance;* that nothing can by possibility be known to the quacksalver and empiric that is not familiar to the educated physician; that a youth of preparation, and a life, however protracted, of ceaseless devotion to his art, are all too little to familiarize him with all the varieties of disease, and the means of meeting them successfully; and that there is no access to the temple of medicine, save through an intimate knowledge of the laws by which we live and move, and have our being.

It is a frequent complaint that the present times, however rapidly they advance in an intellectual point of view, still fall short, physically and morally, of what they ought to be; that mankind are obnoxious to many more diseases now than formerly. There is much that, on a hasty survey, seems to countenance such complaints; in especial, the excessive refinement of manners, and the luxuries attendant on civilization; whence effeminacy and debility—the swelling nomenclature of diseases, and the endless variety of means of cure. The authors of this volume consider such a view, however, as wholly without foundation. They undertake to shew that, with the increase of civilization, the sanatory condition of states and smaller communities has undergone an actual improvement; that diseases, on the contrary, have rather been falling off in number, and decreasing in intensity; and that every onward step in the path of knowledge and true refinement has had a beneficial influence on the entire corporeal being of mankind. It cannot be denied, that, with the progress of civilization, not only does population in general increase, but that the length of individual life is augmented, whilst the liability to sickness, and to the sufferings to which every individual born is obnoxious, are lessened. Epidemical diseases, formerly regarded as necessary evils, and inseparable from humanity, are known within civilized nations only by name. Though perhaps disposed to consider that a life spent in tilling the ground, in fishing, and in hunting, must afford the greatest number of hours for undisturbed enjoyment, still we must draw the distinction between that intercourse with nature which is taken as pastime, and that which is taken as a means of supporting life. The peasant, the fisherman, and the hunter have other tales to tell beside those connected with pleasure and felicity. In the absence of all occupation for the higher faculties the soul dreams on but too readily in a slumbering or half-waking state. To real, to perfect health, harmony of corporeal and spiritual aptitudes is indispensable. The cultivation of higher powers is not necessarily coupled with any thing that is pernicious. The requirements of society, so often opposed to reason, are constant causes of a more passing or more permanent interruption of the sense of well-being; but with a little prudence and reason, the legitimate fruits of good education, the prejudicial influences of such circumstances may be greatly diminished, or entirely superseded. In virtue of the support derived from cultivated intellect, man becomes capable of giving successful battle to all external influences that tend to its detriment—the enlarged views engendered under the influence of social co-operation, tend to arouse the corporeal energies. Good sense and moral equilibrium present themselves as the means best adapted for achieving elasticity under the sorest bodily afflictions. Some travellers, who have lived long among uncivilized people, speak of but few diseases as prevalent among them. The authors ask, and very justly ask, are those rarities real? is not the reason to be sought rather in the inhumanity of the natives, in some sort commanded by necessity, and sanctioned by custom, and in the insufficiency of the remedial means with which they are acquainted? It is difficult to conceive a life similar to that said to have been led by man in his earliert state, as either peculiarly pleasant in itself or advantageous to health. The olden poets tell us that the first races of men knew nothing of disease; this is somewhat like the assertion that before the Fall the earth was without poisonous plants, and the rose without thorns. We find another of the poets of antiquity with much more truth, ranking it among the blessings conferred by Prometheus on primitive men, that he taught them physic—

———————"when prostrate with disease,
And means were none of cure,—no quick'ning drink,
No soothing balm, nothing but death before them—
'Twas then they learned of me the art to draw
The healing potion from the leaf and root."

Though it must be admitted that many of the usages and habits, many of the apparently inevitable and prejudicial influences of our present social state, are the results of refinement and civilization, the means of meeting and confining them within narrow bounds are developed in like and even in greater proportion.

With respect to the question whether has insanity increased under the influence of high mental culture, the authors truly observe, that to regard the culture of the mental powers at large, or of one or more among them, as a ground of their derangement or destruction, is a somewhat hasty procedure. It is not culture, but half-culture that has a pernicious influence upon the mind. The more numerous and the better the educational institutions of a country are, the less numerous the insane. The more the whole of the mental faculties are brought into play, the more certainly will imperfections be set aside. Inaction occasions derangement more frequently than activity. How rarely do we see men of letters who labor in peace and due measure become insane! It is not even intense application of the higher faculties that overthrows the mind, but passion and the vicissitudes of life, against which elevation of soul supplies the truest remedy. Whether the relative number of insane persons is actually greater now than it was in former times, cannot be precisely ascertained. Probably the increased attention now paid to the insane, the effect of the progress of civilization, makes the number of such patients appear greater than they did at former times, when the subjects of insanity were commonly concealed in the private parts of dwelling-houses, on various pretexts; now, to conceal the family misfortune—the disgrace as it was held—and again to escape public interference with relatives. In the present day insane persons are almost invariably placed in establishments especially destined for their reception. The more we advance in our knowledge of insanity, the greater the number of forms which it assumes do we distinguish. We must not infer from this, however that the same diversity did not exist formerly. On the contrary some shapes of mental aberration indicated by our predecessors seem to have disappeared altogether, others to be becoming rarer and rarer. With respect to the deaf and dumb, civilization certainly cannot be charged with having had any share in causing this abnormal condition of the senses; far otherwise, the sole alleviation for the evil that can be had, comes from her hand. The deaf and dumb were formerly a heavy burden on society. With the exception of a very few favored by circumstances, the great majority were left to their own incapacity, to the unmitigated wretchedness of their isolation, in a state of moral and physical degradation. How different at the present time, when brought up and educated in pubiic institutions devoted to the purpose, instructed in reading and writing, their understanding is enlightened, means for communicating with the world around them are supplied, and a substitute is found them for their mute and unavailing organs of hearing and of speech! The same may be said of all the establishments for the blind, the deformed, the halt and the lame.

It would be easy to prove that the improved civilization of the present day is distinguished not only by seeking to remedy corporeal and mental evils by every means at command, but also by its unceasing efforts to destroy the very germs of disease. Commencing with infancy, we see the present time distinguished by an increasing attention to the physical wants of that state, and a diminution of its mortality. The solicitude commences even before children see the light, and is active the moment they do so; the relations between nature and art in the process of parturition are now better understood than formerly; well-timed interference is constantly saving the threatened life of both mother and child—the necessity of proper nursing is now much better understood. Much also has been done to guard against the temptation to commmit child-murder. In the education of children regard is now had not merely, as formerly, to the mental qualities, but to the bodily powers also; and when predisposition to disease exists, judicious means are employed to repress its growth, and eradicate its seeds.

Great improvements have been made in the dress of the community, though something still remains to be done with respect to the female portion of it—all those articles that formerly interfered with the free play of the organs are falling every day into greater discredit. Cleanliness is now much more attended to than formerly—the important influence of the skin on the health of the entire system is now felt and appreciated, as it deserves. The necessity of free ventilation and wholesome air is duly acknowledged, and every means are now adopted to secure these advantages by wide streets, sufficient sewerage, and the discontinuance of sepulture amid the dwellings of man. The facilities for procuring wholesome food are now much increased through the progress of agriculture. The cultivation of the potato, of fresh vegetables, and of various kinds of useful fruit, has an undoubted iufluence on the health of communities. We fully concur with Dr. Willis in deprecating the barbarism of regarding sugar as a luxury, and making it a source of revenue; it being one of the prime necessaries of life, ought to be free as air; the first act of the stomach upon the amylaceous principle, which constitutes about four fifths of our ordinary food, is to turn it into sugar—a fact from which the value of sugar, as an article of nourishment, may be inferred. Adulterations of articles of food are now much rarer than formerly.

Great improvements have been made in the fabrication of kitchen utensils. The sale of poisonous articles is more restricted than it was, and is in far better hands. Toxicology is better understood. Another important feature in modern civilization is the care that is taken of the poor and helpless. The experiment of home-colonization seems to promise permanentimprovement and advantage to the human family. The improved construction and police of prisons is another important point. These are now not merely places of detention and punishment, but schools of improvement also. In the military service, harshness and severity are every day yielding to humane and reasonable treatment. Attention to the wants and comforts of the soldier have now gone far in rendering him, on home service, the healthiest man in the community. The same may be said with respect to the naval service. The scientific study of the diseases of artizans and laborers in detecting the hidden sources of their sufferings, has exposed the means of removing them, or rendering them nugatory. The physician and philosopher working hand in hand here, good fruits have certainly not been wanting. The care taken of the sick poor in hospitals also contributes very much to repress mortality. Contagious diseases have of late years lost much of their virulence, first, from the care with which precaution and prevention are enforced, and then from the care bestowed in exposing and airing, in washing, in heating, and if necessary, in burning suspicious bales and articles in which infection may be suppossed to lurk. These measures, it is probable, have renderedz ymmotic influences inoperative now, that in former ages proved pestilential. These measures also may have caused such diseases as plague and yellow fever, which were contagious in former times, to be contagious no longer. The discovery of chlorine has placed a powerful weapon in our hands against contamination. The mode of treatment now adopted in the treatment of the eruptive diseases, more especially small-pox, has contributed much to diminish their fatality. The dread of cool air is happily overcome; the nursery and sick-room are kept ventilated. The larger and more commodious houses of modern times, in additon to the better clothing and food of the community, prevent the spread, as well as the production, of diseases. With respect to the circumstance of residing in town and country, it must be allowed that a life passed in the open air conduces to health and strength of body; but when to this is added excessive toil, much of the beneficial influence of a country residence is lost. It is therefore indubitable, that the simple natural state, as it has been called, is less favorable to longevity than the civilized condition.

It is worthy of remark, that in England, where unquestionably the greatest amount of material comfort prevails among the community at large, the greatest mean duration of human life, namely, 38 years, also occurs: in Russia it is but 21 years; the comfortable man not only lives better, he lives longer. The great improvements made in diagnosing and treating diseases since the commencement of the present century has contributed a considerable share to their diminution and removal. Percussion and auscultation have done wonders in enabling us to detect with precision and to treat with success the various diseases of the thoracic viscera. The old mode of treating syphilitic diseases was often as fatal in its effects as those maladies themselves. Deformities and imperfections, formerly the prey of ignorant empirics, have been made the subject of study by educated men, and are now removed and remedied by appropriate operations. It surely cannot be said that this or that particular disease has increased in frequency, when it is acknowledged that a much larger proportion of mankind, by attaining to old age, are made obnoxious to its attacks. Where there are few or no subjects for apoplexy to attack, there are few or no apopletic attacks. Civilization can only guard against and abate circumstances that induce disease; it has no power to bestow physical immortality. Precisely in the ratio of the greater mass of life and living energy that presents itself in the civilized world, is the glory of the victory that is won over the multiplied and infinitely various causes which threaten derangement and destruction. References to historical and statistical accounts of almost all diseases satisfy us of the truth of this position. The authors here adduce examples of particular diseases, as phthisis, scrofula, rickets, syphilis, various neuroses, and the phlegmasiæ, in order to prove their statement, namely, that the progress of civilization has materially contributed to the decrease of diseases; in doing which they have succeeded in a manner calculated to produce conviction in every rational mind.

We must, however, discontinue our analysis here, referring the reader to the volume itself. We again repeat that Dr. Willis has done good service to the community by the publication of this truly interesting volume, the value of which has been considerably enhanced by the excellent notes which he has annexed.—*Medico-Chirurgical Review.*

